# Determination of cGMP levels in rodent tissues following oral dosing of a soluble guanylate cyclase stimulator

**DOI:** 10.1186/2050-6511-14-S1-P54

**Published:** 2013-08-29

**Authors:** Nisha Perez, Christopher Graul, Peter Germano, Erik Solberg, Samuel Rivers, Robert Solinga, Joel Moore, Gerhard Hannig, Ada Silos-Santiago, Robert Busby, Daniel Zimmer

**Affiliations:** 1 Ironwood Pharmaceuticals, Cambridge, MA 02142, USA

## Background

**Figure 1:**
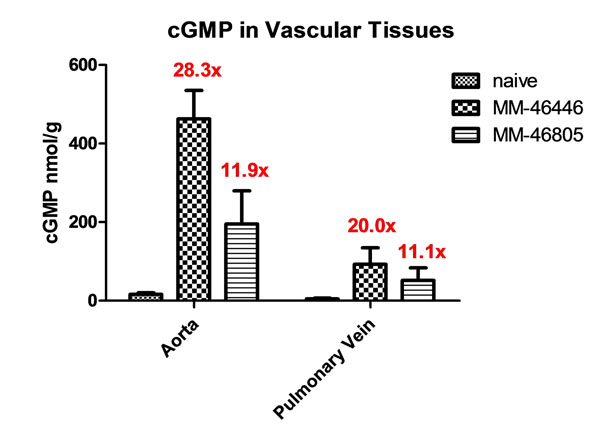
cGMP in vascular tissues

In the vasculature, nitric oxide (NO) binds and activates smooth muscle soluble guanylate cyclase (sGC), leading to increased intracellular cGMP, which triggers smooth muscle relaxation and vasodilation. sGC stimulators are a class of small molecule allosteric modulators, which stimulate cGMP production independently of NO but also act in synergy with NO. Evidence to date suggests that sGC stimulators may be balanced vasodilators, meaning that they elicit vasorelaxation in both the arterial and venous vasculature; however, there have been conflicting reports [1,2]. Our approach to developing a better understanding of the arterio/venoselectivity of sGC stimulators was to measure cGMP concentrations in arteries, veins, plasma, and other tissues of rats orally administered with one of our sGC stimulators (MM-46446 or MM-46805) at a pharmacologically active dose. Results were treated with either vehicle or an sGC stimulator and cyclic GMP levels were measured utilizing an extraction method paired with a sensitive LC/MS-MS detection method (LLOQ = 0.3 nmol). This cGMP quantitation method is linear from 0.3 – 2,890 nmol with no need for either dilutions or derivatizations, unlike current ELISA based methods that are linear from 0.2 – 3 nmol and require acetylation plus sample dilutions. Using this cGMP detection method, we were able to determine that basal cGMP levels appear higher in the aorta when compared to the pulmonary vein, 16.4 nmol/g versus 4.64 nmol/g, respectively. Upon treatment with either of the two sGC stimulators, aorta cGMP levels increased 28.3 and 11.9-fold, respectively, and pulmonary vein cGMP levels increased 20.0 and 11.1-fold, respectively (Figure 1).

## Conclusion

The current study shows that our sGC stimulators are able to increase cGMP levels in both the rat aorta and pulmonary vein and indicates the potential for sGC stimulators to act as balanced vasodilators.
